# Building a Community-Academic Partnership: Implementing a Community-Based Trial of Telephone Cognitive Behavioral Therapy for Rural Latinos

**DOI:** 10.1155/2012/257858

**Published:** 2012-09-19

**Authors:** Eugene Aisenberg, Meagan Dwight-Johnson, Mary O'Brien, Evette J. Ludman, Daniela Golinelli

**Affiliations:** ^1^School of Social Work, University of Washington, 4101 15th Avenue NE, Seattle, WA 98105, USA; ^2^West Los Angeles Veterans Affairs Medical Center and David Geffen School of Medicine, Department of Psychiatry and Biobehavioral Sciences, University of California at Los Angeles, Los Angeles, CA 90095, USA; ^3^Rand Corporation, 1776 Main Street, Santa Monica, CA 90401-3208, USA; ^4^Yakima Valley Farm Workers Clinic, Behavioral Health Services, 918 E. Mead, Yakima, WA 98902, USA; ^5^Center for Health Studies, Group Health Cooperative Research Department, 1730 Minor Avenue, Suite 1600, Seattle, WA 98101, USA

## Abstract

Concerns about the appropriate use of EBP with ethnic minority clients and the ability of community agencies to implement and sustain EBP persist and emphasize the need for community-academic research partnerships that can be used to develop, adapt, and test culturally responsive EBP in community settings. In this paper, we describe the processes of developing a community-academic partnership that implemented and pilot tested an evidence-based telephone cognitive behavioral therapy program. Originally demonstrated to be effective for urban, middle-income, English-speaking primary care patients with major depression, the program was adapted and pilot tested for use with rural, uninsured, low-income, Latino (primarily Spanish-speaking) primary care patients with major depressive disorder in a primary care site in a community health center in rural Eastern Washington. The values of community-based participatory research and community-partnered participatory research informed each phase of this randomized clinical trial and the development of a community-academic partnership. Information regarding this partnership may guide future community practice, research, implementation, and workforce development efforts to address mental health disparities by implementing culturally tailored EBP in underserved communities.

## 1. Introduction

 Despite substantial efforts by researchers, policy makers, and federal funding sources to improve access to mental health care for ethnic minority patients, disparities in access to, quality of, and outcomes of mental health interventions persist [1–4]. For example, Latinos suffer a disproportionate burden of disability from depression compared to Whites [5] because they are less likely to receive depression treatment and the treatment they do receive is often of poorer quality compared to treatment received by Whites [6–8]. In research studies, implementation of culturally tailored evidence-based practices (EBPs) has been shown to reduce disparities [9, 10]. However, there is little evidence that dissemination of EBPs into real-world practice settings has substantially reduced disparities in the access and utilization of mental health services across many racial and ethnic minority groups [11, 12]. 

 Several reasons have been identified as contributing to the muted impact of evidence-based interventions within underserved communities of color: (1) the lack of inclusion of ethnic minority participants in randomized clinical trials (RCTs) which serve as scientific gold standard of effective practice, (2) the failure to include communities of color and the health systems that serve them in the processes of defining, implementing, and evaluating EBPs, (3) the dearth of ethnic minority researchers within academia, (4) the shortage of ethnic minority mental health providers to deliver EBPs, and (5) the lack of responsiveness to cultural context and norms of ethnic minority communities within the content and structure of the EBPs themselves [13]. 

Historically, persons of color have been substantially underrepresented in RCTs. The U.S. Department of Health and Human Services (2001) report, *Mental Health: Culture, Race, and Ethnicity. A supplement to Mental Health: A Report of the Surgeon General, *examined controlled clinical trials used by professional associations and government agencies to establish treatment guidelines for four major mental health conditions (bipolar disorder, schizophrenia, depression, and ADHD) and to form the science base for evidence-based practices [5]. From 1986 to 1994, nearly 10,000 subjects participated in RCTs evaluating the efficacy of interventions for the aforementioned disorders; in total, only 561 African Americans, 99 Latinos, 11 Asian Americans/Pacific Islanders, and 0 American Indians/Alaskan Natives were identified [14, 15]. This lack of inclusion of adult ethnic populations is similarly found among 27 studies from 1986–1997 forming the evidence base for the American Psychiatric Association guidelines for depression care. Among the nearly four thousand participants in these studies, there were only 27 African Americans, 2 Asians, and 241 nonwhite participants [16]. The failure to include meaningful numbers of persons of color in RCTs makes it unclear whether these EBPs are effective in these populations. Furthermore, if RCTs of EBPs are not conducted with ethnically diverse participants, they have typically been normed or standardized void of cultural context and realities, thus challenging the ability of the EBP to later be successfully implemented among diverse populations [13]. 

“The development of evidence-based practice strategies, a hallmark of clinical-services research, typically starts with development and evaluation of treatment and service-delivery interventions by researchers without strong community participation” [17]. This linear top-down approach—with knowledge generated in academic settings with highly trained specialist providers—then transferred to community practitioners who often work in systems with limited resources may have limited application to community practice settings with diverse patient populations. Even within academia, there is a dearth of ethnic minority researchers, especially as principal investigators. This scarcity contributes to a dominant culture and outsider perspective in engaging communities of color in research and clinical trials rather than a culturally informed perspective and understanding of the context of such communities that seek meaningful change in the availability and delivery of mental health services. 

Other contributing factors that thwart the distribution of treatment to address disparities include the lack of linguistically and culturally responsive mental health providers to deliver quality mental health services [18] and the lack of opportunities and funding for community partners to be trained in EBPs. Related to these factors is the reality that ethnic minority patients are often served in safety net health care systems that often lack resources to implement and sustain those culturally appropriate EBPs that do exist. Also, few community providers and practices have the requisite resources and research infrastructure to evaluate the effectiveness of their community-developed, culturally-informed practices in order to have them considered to be evidence-based. As a result, most evidence-based practices on the various Federal lists that may have any applicability to communities of color were not designed specifically for those communities [19]. Use of scarce resources to implement evidence-based practices that do not appropriately address the needs and cultural values of ethnic minority patients or the resource limitations of the clinical settings in which they are served may perpetuate disparities in care, as patients may not enter or remain in care, and health systems may not be able to sustain them [13, 20].

In 2004, the Institute of Medicine's Clinical Research Roundtable recommended the promotion of public participation and community partnership in all phases of research to increase the relevance of clinical research and promotion of research findings in ethnic minority populations [21]. Community-based participatory research (CBPR) and a variant, community-partnered participatory research (CPPR), are two approaches seeking to address the identified shortcomings of traditional research and dissemination methods [17, 22, 23]. Both approaches emphasize the active participation of community members, specifically, the inclusion and engagement of diverse community stakeholders in meaningful and equitable power-sharing and collaboration in all phases of research. A major difference between these inclusive approaches is their understanding of community members. Whereas CBPR models empowerment and leadership of grassroots community members, CPPR relies on community agencies as brokers for community members and emphasizes the adaptation of evidence-based practices [17]. 

In this paper, we highlight the processes of development and engagement in a community-academic partnership to adapt and pilot test an evidence-based practice demonstrated to be effective for urban, middle-income, English-speaking primary care patients with major depression and translated and culturally tailored for rural, low-income, Latino (primarily Spanish-speaking) primary care patients identified with major depressive disorder. We successfully completed a randomized pilot test of telephone-based cognitive behavioral therapy (CBT) for depression in a primary care site in the Yakima Valley Farm Workers Clinic (YVFWC), a network of 17 community health centers serving primarily low-income Latino patients in rural Eastern Washington and Oregon. We have reported previously the clinical outcomes of the randomized trial in which one hundred adults, including twenty men, with major depression were recruited from a rural primary care practice and randomized to an eight-session manualized telephone CBT intervention delivered by bilingual social workers versus care as usual [24]. In brief, in intent-to-treat analyses, patients randomized to CBT by phone were more likely to experience improvement in depression scores over the six-month follow-up period compared with patients randomized to usual care (*β* = −.41, *t* = −2.36, df = 219, *P* = .018, for the SCL; *β* = −3.51, *t* = −2.49, df = 221, *P* = .013, for the PHQ-9). A greater proportion of patients in the CBT group than in the usual care group achieved treatment response at three months (*P* = .017), as indicated by a 50% improvement in SCL depression score or a score <.75, and reported high satisfaction with treatment (*P* = .013) [24]. Throughout the planning and implementation phases, this study was informed by the values of CBPR, nevertheless, our community-academic partnership more accurately reflects the CPPR model. 

## 2. Methods

### 2.1. Setting and Study Procedures

This study was approved by the Human Subjects Committees of the UW and the YVFWC. In 2008, the study site, the Family Medical Center in Walla Walla, WA served 8,559 unduplicated medical/pediatric patients; of these 53.3% Latino, 44.4% Spanish-speaking, 20.7% Seasonal Farm Workers, 6.5% Migrant Farm Workers, and 30.7% uninsured. 

### 2.2. Recruitment

 The study employed 3 part-time bilingual, bicultural recruiters hired from the local community and from existing clinic staff. Trained by the academic investigators and clinic administrator, these recruiters approached patients in the waiting room and asked if they would like to hear about a depression research study; if they agreed, patients were taken to a private area near the waiting room for verbal informed consent and screening. Clinic providers could also refer patients for screening. Adult patients were study eligible if they had a primary care provider (PCP) in the clinic, self-identified as Latino, spoke English, or Spanish, and screened positive for current major depressive disorder. Patients were excluded if they screened positive for bipolar disorder, cognitive impairment, current or lifetime psychotic symptoms or disorder, current substance abuse, or acute suicidal ideation. Following discussion of study procedures and written informed consent in the patient's preferred language, enrolled patients completed baseline surveys. Patients were then randomly assigned to receive the eight-session, manualized telephone cognitive behavioral therapy intervention or usual care (UC). Randomization was stratified by referral source and gender. All patients were asked to complete 3- and 6-month follow-up surveys to assess clinical characteristics and depression outcomes. 

Qualitative data was obtained over the telephone by trained interviewers from patients who received the CBT intervention and from primary care physicians at the clinic site. Each patient randomized to the CBT intervention was asked to complete a semistructured qualitative exit interview 6 months after baseline. This interview sought to elicit the patient's perspective and experience regarding their satisfaction with the intervention, sociocultural appropriateness of the treatment, and barriers to treatment adherence. After all study patients completed treatment, five primary care physicians at the clinic site were asked to complete a qualitative interview via the telephone. The provider interview sought to elicit provider opinion about: the care their patients received, their interactions with the therapist, benefits of the treatment to their patients, barriers to study treatment, and additional components or services needed.

Semistructured interviews followed well-established procedures and consisted mostly of open-ended, qualitative questions but also included some close-ended questions [25, 26]. At the beginning of each interview, interviewers began with what Spradley (1979) calls the “grand tour question” and asked each participant to describe in their own words their experiences with the program [27]. Trained interviewers used nonspecific prompts (e.g., “can you tell me more?” “can you elaborate?”) to encourage participants to be as detailed as possible. This broad (and intentionally) undirected questioning allowed participants to indicate what aspects of the program were most salient to them and why. Open-ended questions were asked before prompts and close-ended questions to minimize bias, allowing interviewers and respondents the opportunity to explore new leads and related topics [26, 27]. Following broad questions, interviewers used standard probes, such as verification and compare and contrast questions. The interviews were audiotaped and transcribed then professionally translated. Bilingual study personnel from at least 2 different Spanish-speaking countries reviewed the translated interviews. They were then back-translated, and all differences resolved through study meetings. 

 Coding of patient and provider interviews was done by a doctoral level research assistant. The lead author separately examined a small number of random transcripts to promote reliability. The qualitative data analysis strategy drew on principles of grounded theory [28], which involves examination of narrative data, searching for patterns and themes that help explain a given phenomenon, and then coding the data to further corroborate or modify themes. Atlas/ti software was used to review each transcript and mark instances of each theme.

## 3. Results

### 3.1. Partnership Development

 One of the academic investigators (EA), who is himself bilingual and Latino, initially contacted YVFWC staff via the University of Washington (UW) Liaison in Yakima, WA. The purpose of the visit was to introduce himself following his arrival in Washington State. He learned that due to earlier experiences, YVFWC personnel were initially reluctant to partner with UW researchers because previous research projects had not resulted in tangible or lasting benefits to YVFWC clinics or patients. Their experience of the traditional paradigm of the researcher coming into the community and collecting data and publishing the findings was oppressive and not mutually beneficial. Following this initial visit, the investigator (EA) travelled to the rural region of Eastern Washington State approximately every six weeks and met with administrative and clinical representatives of the YVFWC. These face-to-face meetings were crucial to build rapport and dispel the legacy of mistrust built by years of noninclusion and non-partnership. After a year of face-to-face meetings in which staff educated him about salient issues for the local rural Latino population, sufficient trust developed that YVFWC staff asked the UW investigator to consult with them regarding the implementation of multi-systemic therapy for adjudicated Latino youth. 

 In the midst of this successful collaboration, YVFWC staff then began expressing concern about untreated depression in their clinics, especially among patients with diabetes. YVFWC staff identified depression as a significant under-addressed problem in part due to a shortage of mental health practitioners and difficulty accessing community mental health centers focused on treatment of severe mental illness. Systematic screening for depression was not conducted in any of the clinic sites, and treatment options were often limited to prescribed medications. Clinic staff acknowledged that nearly fifty percent of all patients identified as suffering depression either failed to pick up their medications or did not take them according to the treatment regimen. This identification of gaps in service by YVFWC was a crucial starting point for the partnership. Rather than being determined by academic researchers and outsiders to the local community, YVFWC staff identified the need and defined the issue to be addressed. They were respected as experts of their experience.

 Soon after, another academic investigator with experience in primary care depression interventions for Latinos (MDJ) arrived at UW. Also, colleagues at the Group Health Research Institute published findings from a trial of telephone CBT [29]. The lead author informed these colleagues about the desire of YVFWC to address depression care. They were very open and committed to partner with the YVFWC and contribute their expertise. UW investigators (EA and MDJ) presented the evidence-based telephone CBT intervention to YVFWC staff as a possible strategy to address barriers to depression treatment such as lack of transportation and limited availability of local Spanish speaking therapists. Later, in-person meetings were held at two YVFWC clinics, with primary care physicians and administrators participating. One of these clinics, which had no onsite mental health services, expressed interest in participating in a pilot trial of telephone CBT. Subsequently, study partners engaged in discussion regarding the design of the proposed study and then the UW investigators and YVFWC staff collaborated in writing and submitting a grant proposal to obtain funding from the National Institute of Mental Health to support such a trial. This randomized clinical trial focused on a study population that has little access to depression care services in the community and is underrepresented in intervention trials. Few Latinos have access to evidence-based psychotherapies (EBPs), especially in primary care where they are most likely to seek depression treatment [7, 8, 30]. 

 Buy-in from the primary care physicians and staff at the selected study site was crucial to the study's outcomes. The study's format of use of telephone to provide depression care allowed for implementation in a rural primary care clinic that lacked onsite mental health specialists, especially bilingual and bicultural ones. The study facilitated engagement with a trained bilingual mental health provider and promoted access to evidence-based depression care to rural Latinos in a region where psychotherapeutic services for depression were nearly nonexistent. These were key features supported by the physicians and YVFWC personnel. Conducting this trial in a primary care setting among low-income rural Latino patients, many with comorbid conditions and competing life priorities also helped address concerns of community providers about the feasibility and acceptability of this intervention in real-world settings and populations. 

A central feature of this phase was a shift from a “research into practice” model to a “research in practice” model in which clients and YVFWC staff partnered with researchers in the generation of knowledge about the effectiveness of the telephone CBT intervention. This paradigm shift required that the history, experiences, and wisdom of people of color along with the expertise of practitioners be valued in much the same way as is the science of efficacy [31]. Consistent with the values of CBPR and CPPR, resources and expertise were shared. YVFWC partners took the lead in hiring study recruiters and study therapists, some of whom were internal to the organization and others who were external to it, and provided ongoing administrative supervision. This level of involvement from the community clinic was especially important given the physical distance between the academic investigators and the community site and allowed the investigators to quickly and safely address clinically urgent situations, such as suicidal ideation detected during recruitment. Local bilingual-bicultural study staff were able to inform academic partners of local values and customs that were incorporated into study procedures, such as recruitment. 

Ongoing and consistent communication with the YVFWC and study site personnel was a key feature of our mutually beneficial partnership. It enhanced shared decision making in addressing important issues such as the recruitment of study participants at the clinic site in a way that was valued by the primary care physicians and seamless with existing clinic practice. It confirmed the importance of the efforts and expertise of community partners and deepened academic partners' understanding of depression as experienced by low-income rural Latinos. In addition, it enhanced the quality of the rigorous research. 

### 3.2. Therapist Training

We trained and supervised five Latino/a bilingual and bicultural part-time therapists ranging from social work students with little clinical experience to an experienced MSW therapist. With upfront training, telephone role play, and weekly supervision novice therapists were able to competently deliver the structured intervention and adapt manual content to address patients' needs. Initial training of study staff in manualized CBT intervention was conducted in person at a designated clinic of the YVFWC network by the academic partners. Nonstudy clinical staff of YVFWC were intentionally invited to participate in this training. This outreach and inclusion were viewed positively by the community partner since it provided concrete benefits to staff and patient populations. Mentoring and training study staff were also a way for academic partners to invest in the community. One study recruiter hired when she was a bachelors' student, later became a study therapist when she enrolled in a Masters of Social Work program. Several study therapists, trained in evidence-based treatment through our pilot study, subsequently gained employment in the community and currently provide important leadership in mental health services in the community and in schools. In both instances, academic partners provided strong letters of recommendation on their behalf.

Further evidence of our strong community-academic partnership was the provision of ongoing supervision to the study therapists. It was a collective responsibility, with representatives from the YVFWC and researchers providing weekly telephone-based supervision to the study therapists. Weekly supervision promoted consistent delivery of manual content, allowed therapists to make changes in practice quickly, supported outreach efforts, and allowed supervisors to monitor patients' clinical status and provide support for crisis management. In general, patients completed the session modules in order, but the schedule was sometimes modified for a few participants—for example, to switch the order of modules or to use two sessions for a single module. 

### 3.3. Intervention Adaptation

 The collaborative efforts to revise the study manual were instrumental in enhancing its usefulness and meaningfulness to participants randomized to the intervention arm of the study. The original English language telephone therapy manual required revision which was done in collaboration with YVFWC clinical staff. It was initially revised by a MSW student and back-translated by the lead author. The text was revised at a 6th-grade reading level in Spanish. Also, the content of examples and vignettes was tailored to rural Latinos, including the use of Latino names, and reflected situations relevant to rural Latinos, including family and parenting themes. An implicit assumption underlying the activity schedules in the original intervention was “taking time for one's self.” Because this individualistic orientation runs counter to an emphasis in Latino culture, especially for women, of putting the needs of family members first, the pleasant activities presented in the original manual were modified to reflect activities that could be engaged with children and family members and are accessible in time and cost to low-income rural Latinos. Bilingual members of YVFWC (some with farm worker backgrounds) reviewed the translated manual to ensure that language, idioms, and vignettes were appropriate for the local Latino context and culture. They made several important suggestions to strengthen the relevance of the vignettes and homework activities for the rural Latino population. Other adaptations included the provision of case management services as needed to assist patients to navigate the health system, address practical barriers, and access community-based resources [32, 33]. In response to the cultural value of *personalismo,* (interpersonal rapport), we modified the original manual to provide the opportunity for the patients to meet the therapist in person prior to engagement in the CBT protocol.

### 3.4. Patient Response

See Table 1 for a description of the participants' characteristics at baseline. The revised study's manual sought to engage Latino patients' understanding of depression and address depression in the context of the patient's culture and life experiences, in particular, the patient's life within the family. In qualitative exit interviews, nearly all participants felt that the stories contained in the manual reflected and captured their lived experiences. They remarked that the stories were useful to them and helped them learn new and relevant skills. Learning skills to identify and change negative thinking was one of the major strengths of the program. One participant described how the therapist encouraged her to write about positive things in her life instead of only focusing on the negative. In this way, the skills from positive thinking transferred to her writing; an activity she already enjoyed. Several participants indicated that they learned new things directly from the manual, including new knowledge about depression.

 Patients reported several additional benefits. Most patients reported that they had developed a trusting relationship with the therapist, regardless if they had an initial in-person session with their study therapist. The vast majority of participants reported very positive feelings about their relationship with their therapist. Therapists were described as “understanding,” “professional,” “patient,” “encouraging,” “comfortable,” “good communicator,” “trusting,” and “easy to relate to.” Several respondents expressed they felt like they really mattered to the therapist. Also, participants rated the ability of their therapist to explain new concepts very high. 

The majority of the clients reported positive strong social support for their participation in the program particularly from family members or friends who knew about their involvement in the program and supported them, for example, helping them complete homework. Patients did not find privacy concerns to be a barrier as most had cordless telephones that allowed them to find a quiet place inside or outside the home for sessions. 

Overall, most participants reported very good experiences with the telephone-based delivery of CBT. Respondents noted that the telephone delivery was “convenient,” “comfortable,” and “private.” Several expressed that they were at greater ease in their own homes and therefore able to speak more freely and openly in the sessions. The telephone broke down barriers for those who were shy or felt embarrassed about participating in the program. This was especially true for the participants who were men—the anonymity provided by the telephone contact enhanced their sense of safe participation and lessened their sense of stigma. In addition, the telephone was convenient for many clients who were balancing hectic work, child-caring and domestic responsibilities since sessions were held at the time that was preferred by the client, even if beyond normal clinic hours. Finally, the use of the telephone for delivery of the manualized intervention addressed the barrier of transportation. Several participants noted that they did not have access to transportation, which would have been an insurmountable barrier if required to come to the clinic for face-to-face therapy. 

In terms of suggestions, several respondents recommended that the manual contains more review of depression as well as a brief summary of the main highlights of each session. Also, a few participants expressed a desire for a longer intervention to address more serious depression or personal problems. 

### 3.5. Primary Care Provider Response

 Qualitative feedback from the clinic's five primary care physicians highlighted the strengths of the telephone-based program and their satisfaction with it. They valued the team's consistent and ongoing feedback and communication with them about patient recruitment and the study's progress. They indicated that the waiting room screening process was efficient and did not delay their appointments with patients. Uniformly, providers expressed strong satisfaction with the participation and retention of clients and the marked improvement of their patients in a relatively short period of time. This success was noteworthy due to the fact that prior to the study it was customary that nearly fifty percent of patients would fail to pick up their medications or follow treatment protocol. Providers made two principal recommendations: (1) to expand the criteria of eligibility to participate in the program to include patients with more severe symptoms, for example, patients with bipolar disorder, and (2) to increase the availability of case management services.

### 3.6. Ongoing Partnership

In this phase, community partners copresented the findings of the pilot study and lessons learned at esteemed regional and national conferences. They collaborated in publishing study findings and shared authorship. Also, they collaborated in additional efforts to secure funding in order to expand the use of the telephone-based manualized intervention across the YVFWC network. This participation promoted leadership by YVFWC representatives in knowledge building and in research. In preparation for future trials, we have further modified the manual to include optional guidelines for involving family members to support behavioral activation and plan to expand case management services to assist some patients to access additional community resources as warranted. The participation of YVFWC representatives was a visible and tangible confirmation of the meaningful and authentic partnership enjoyed by our community and academic partners. 

## 4. Conclusion

 This study adds to the scant empirical research regarding the adaptation of EBTs to promote their fit for specific ethnic communities by addressing: (1) lack of representation of persons of color in RCTs, (2) lack of representation of service settings that serve communities of color in the development and testing of EBPs, (3) the lack of ethnic minority academic researchers leading RCTs, and (4) resulting lack of representation of cultural values for persons of color in the EBPs themselves. Our study comprised of 101 low-income Latino patients, 20 of whom were male. Our experience highlights how an authentic community academic partnership can promote successful implementation of effectiveness trials and begin to address existing disparities in the access and utilization of mental health services. 

Engagement with community partners was valued by researchers and deemed essential in the successful implementation of the telephone CBT program. Our experience illustrates that listening well is crucial to developing a trusting relationship between academic and community partners. Such efforts should be initiated before pursuing specific goals. Allowing the community partner to identify unaddressed needs also promotes community investment in the successful outcomes.

Community-academic partnerships are not always easy to develop and maintain due, in part, to historical mistrust arising from the practice of academic researchers who gather and publish their data but fail to leave tangible benefits for the community or the collaborating community-based agency. It is crucial that the community partners engage in meaningful and authentic ways. Researchers must engage communities of color as legitimate partners in the pursuit of advancing knowledge and transforming the provision of mental health systems of care and services. Acknowledging the lack of ties between the community and researchers, the National Institute of Mental Health in its 2006 report, *The Road Ahead*, called for such collaborative and sustainable partnerships among diverse stakeholders [34]. Such partnerships need to ensure community participation and cultural tailoring for successful intervention development and promotion of quality of care. Key characteristics of successful partnerships involving community-based agencies and academic institutions include shared decision making, equitable sharing of resources and power, and mutually beneficial goals and reciprocity [35]. Meaningful inclusion of communities of color at the table in the definition, planning, development, and dissemination of EBP that is culturally responsive with regards to cultural, linguistic, familial, and unique mental health service needs is crucial. Further research regarding the development and effectiveness of community-academic partnerships is clearly warranted.

The results of our randomized trial [24] suggest that telephone-based CBT is effective in reducing depressive symptoms among rural Latino primary care patients, and the qualitative results described here show its great promise to effectively address many of the known sociocultural barriers to treatment in this population [36–45]. It fostered engagement with patients who might not be reached by traditional in-person treatment as it eliminates travel, waiting time, and allows for more flexible scheduling, even beyond normal clinic hours. In rural settings where access to psychotherapists is limited telephone intervention may allow treatment by therapists in a different location. Because the stigma attached to visiting a mental health provider may be greater in small rural communities where anonymity is not characteristic and privacy is a concern, telephone treatment can provide more confidential treatment. Also, telephone intervention may have an advantage over clinic-based intervention as it can allow for “*in vivo*” instruction and modeling to the client in the home environment as issues surface during the phone intervention and are immediately addressed. In addition, use of the telephone to provide weekly supervision enables sharing of scant resources as supervisors do not need to reside in the same locales as the study therapists.

Our experience and the literature on implementation of EBPs [11, 46] corroborate the need for workforce development to implement and sustain EBPs in primary care settings serving Latinos. Given the limited supply of licensed practitioners and the vast need for bilingual and culturally responsive services, it is crucial to support the development of community-academic partnerships and increase their number. Such efforts require increasing the number of ethnic minority students entering the behavioral health professions, as they are more likely to practice in communities of color, to be bilingual, and to be culturally informed [47]. To date, such efforts have focused on bringing ethnic minority students to the academy, often distant from their home communities and sources of social support. New initiatives are warranted that bring the academy to the community to promote skill development of practitioners who are committed to addressing the mental health needs of their communities. Rather than requiring rural practitioners to come to a distant university, it is crucial to provide training in the rural community allowing trainees to learn from local and national experts while maintaining connections to their culture, context, and systems of support. Increasing the number of bilingual, bicultural therapists in the community will support dissemination of established EBPs and create opportunities for developing and testing culturally tailored EBPs *within *diverse communities. Concrete support for the development of authentic community-academic partnership is thus paramount since it takes substantial time and effort. The investment of time to build an effective partnership is not often valued by the academy, particularly if one is seeking to gain tenure. Without active support, scholars seeking to engage in the development of community-academy partnership will continue to face a potent barrier. To engage communities of color and to enhance the provision of culturally competent mental health services policy makers and practitioners must listen to and learn from these communities and their contextual realities. Information regarding our partnership and our recognition of the importance of the culture, context, and environment of rural Latinos may guide future community practice, research, implementation, and workforce development efforts to address behavioral health disparities by implementing culturally informed EBPs in underserved communities.

## Figures and Tables

**Table 1:** Characteristics at baseline of 101 patients who received telephone-based CBT or enhanced usual care.

Variables/Category	Overall *N* = 101		Intervention *N* = 50		Usual care *N* = 511	
	*N*	%	*N*	%	*N*	%
Age						
Mean + SD	39.81 ± 10.56		41.17 ± 9.69		38.54 ± 1.27	
Female	79	78	39	39	40	78
Nativity						
USA (excluding Puerto Rico)	4	4			4	8
Mexico	92	91	47	94	45	88
Other	5	5	3	6	2	4
Primarily Spanish speaking	84	84	43	86	41	82
Education						
<6 yrs	27	27	14	29	13	26
6–11 yrs	50	50	24	49	26	51
HS graduate	14	14	7	14	7	14
Some college or higher	9	9	4	8	5	10
Employed full/part time	50	50	26	53	24	47
Uninsured	42	41	16	32	26	51
Married	62	62	31	63	31	61
Annual household income						
≤$5000	8	9	2	2	6	14
$5001–$15,000	36	40	23	48	13	30
$15,001–$25,000	31	34	16	33	15	34
≥$25,000	17	19	7	15	10	23
Agricultural worker status						
Migrant worker	10	10	7	14	3	6
Seasonal worker	32	32	15	30	17	33
Baseline SCL depression scale score^1^						
Mean + SD	1.79 ± .77		1.77 ± .77		1.81 ± .78	
Anxiety disorder	58	57	23	46	35	67
Probable alcohol or substance disorder	13	45	5	39	8	50
>3 medical problems	30	39	17	44	13	33

^1^Hopkins Symptom Checklist—scores range from 0 to 4 with higher scores indicating more severe depression.
